# CO_2_ Gap Alone Is Not a Prognostic Marker for 28-Day Survival of Patients Undergoing a Transcatheter Aortic Valve Replacement

**DOI:** 10.3390/jcm14134612

**Published:** 2025-06-29

**Authors:** Lisa Thiehoff, Julia Alexandra Simons, Steffen B. Wiegand, Gereon Schälte, Jörg W. Schröder, Anna Fischbach

**Affiliations:** 1 Department of Anesthesiology, University Hospital Aachen, 52074 Aachen, Germany; lisa.thiehoff@rwth-aachen.de (L.T.); alexandra.simons@rwth-aachen.de (J.A.S.); gschaelte@ukaachen.de (G.S.); jschroeder@ukaachen.de (J.W.S.); 2 Department of Anesthesiology and Intensive Care Medicine, Hannover Medical School, 30625 Hannover, Germany; wiegand.steffen@mh-hannover.de; 3 Department of Anesthesiology, Intensive Care and Emergency Medicine, Hermann-Josef Hospital Erkelenz, 41812 Erkelenz, Germany

**Keywords:** venous-to-arterial carbon dioxide difference, transcatheter aortic valve replacement, CO_2_ content, ratio of venous-to-arterial CO_2_ difference to arterial-to-venous O_2_ content

## Abstract

**Background:** The venous-to-arterial difference in partial pressure of carbon dioxide (CO_2_ gap) has been suggested as a marker of cardiac output and clinical outcomes. This study aimed to evaluate the CO_2_ gap as a prognostic indicator for 28-day survival in patients undergoing transcatheter aortic valve replacement (TAVR) and to explore its relationship with cardiac function and lactate levels. **Methods:** In this prospective cohort study, 50 TAVR patients were stratified based on their left ventricular ejection fraction (LV-EF) and survival status. Central venous and arterial blood samples were collected at five time points to measure blood gas parameters. The primary endpoint was the prognostic value of the CO_2_ gap for 28-day survival. Secondary endpoints included group differences in the CO_2_ gap, its correlation with lactate levels, and CO_2_ content analysis. **Results:** ROC analysis indicated limited prognostic value for 28-day survival. The CO_2_ gap was higher in non-survivors than in survivors (11.1 mmHg vs. 6.8 mmHg, *p* = 0.039), but showed no significant difference between individual time points. The CO_2_ gap between cardiac (LV-EF ≤ 50%) and non-cardiac (LV-EF > 50%) groups showed no significant difference. Lactate and CO_2_ gap showed no correlation, except at T2 in the cardiac group (*p* = 0.039, r = 0.525). CO_2_ content showed no significance, except at T5, where it was significantly higher in survivors (5.3 mL/dL vs. 1.1 mL/dL, *p* = 0.003). **Conclusions:** The CO_2_ gap did not emerge as a reliable prognostic marker for 28-day survival in TAVR patients. Further studies are needed to explore its clinical relevance.

## 1. Introduction

Aortic valve stenosis is the most prevalent form of valvular heart disease, affecting approximately 2 to 4% of individuals over 75 years [1]. If left untreated, this condition is associated with a significantly increased mortality [2]. In consequence, valve replacement should be considered once the disease becomes symptomatic, if left ventricular function becomes impaired despite the patient remaining asymptomatic, or if criteria such as severe valve calcification are met [3]. Studies have demonstrated that transcatheter aortic valve replacement (TAVR) is a suitable alternative to surgical aortic valve replacement (SAVR) in patients with a high surgical risk [4,5] and demonstrates comparable efficacy in patients with an intermediate [6,7] and low risk [8,9,10,11] profile. Despite advancements in TAVR techniques and improved procedural outcomes in recent years, this method remains associated with notable risks, including high mortality and procedure-related complications [4,5,7,12]. For patients undergoing elective TAVR, the reported 30-day mortality rate is 1.1%, which rises to 3.7% in non-elective cases [13]. Accurate risk stratification and prediction of patient outcomes remain essential for optimizing treatment decisions. However, current methodologies for individual risk assessment are still limited, emphasizing the need for further research to improve clinical decision-making.

The central venous-to-arterial difference in partial pressure of carbon dioxide (CO_2_ gap) serves as a valuable parameter for assessing the adequacy of systemic blood flow in eliminating carbon dioxide produced by tissue metabolism [14,15]. This measurement can be obtained using either a mixed venous or central venous blood sample and an arterial blood sample to calculate the CO_2_ gap [16,17]. Research has demonstrated a significant correlation between the CO_2_ gap and cardiac output [14,18,19]. An elevated CO_2_ gap has been identified as a reliable indicator of low cardiac output. Notably, patients experiencing septic shock and impaired cardiac function have exhibited higher CO_2_ gap values, which were associated with increased 28-day mortality compared to patients with preserved cardiac function [18]. Furthermore, in patients undergoing high-risk surgical procedures, an increased CO_2_ gap at the time of intensive care unit (ICU) admission has been associated with a higher incidence of postoperative complications [20]. Similar findings were reported by Futier et al. [21], who investigated the potential role of the CO_2_ gap in guiding goal-directed therapy. Their study concluded that patients who developed complications following high-risk surgery presented with significantly higher CO_2_ gap values than those without complications.

Despite the current body of evidence, however, the precise role of the CO_2_ gap in predicting outcomes in patients undergoing TAVR remains unclear.

This study aims to assess the potential of the CO_2_ gap as a prognostic marker for 28-day survival in patients undergoing a TAVR and exploring its relation to cardiac function and tissue perfusion.

## 2. Materials and Methods

In a prospective trial, we analyzed clinical data of 50 patients who underwent a TAVR between February and June 2024 at the RWTH Aachen University Hospital, Germany. All patients provided written informed consent. The study follows the analytical approach previously described by Muller et al. [18].

This study was approved by the Ethical Committee of the RWTH Aachen University Faculty of Medicine (EK23-338, date of approval: 11 December 2023) and was registered in the German Clinical Trials Register (DRKS), registration number DRKS00033047.

As the present study was designed as a pilot study, no formal power calculation was conducted. At the time of protocol development, insufficient data were available to allow a reliable estimation of the expected effect size. The primary objective was to explore potential associations and to generate preliminary data that may guide the design of adequately powered future studies.

### 2.1. Patients

**Figure 1:**
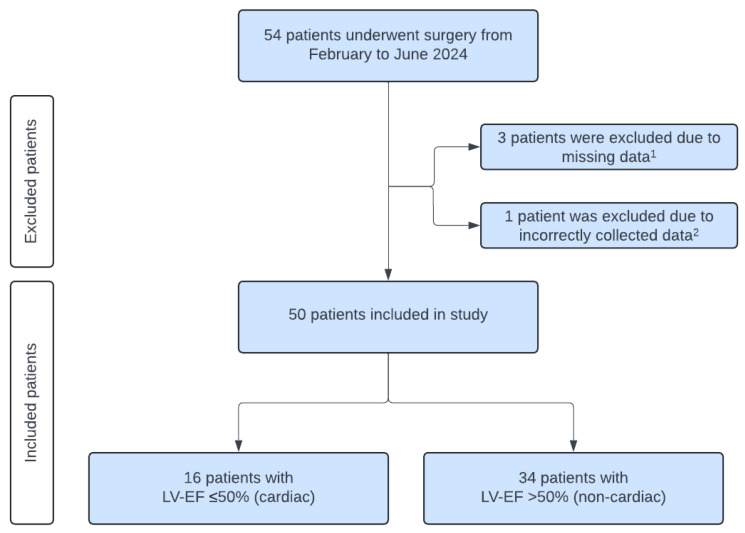
Patient inclusion chart. LV-EF = left ventricular ejection fraction. ^1^ A blood sample could not be taken at T5 for one patient, and for two patients, no blood samples could be taken from T3 to T5 because complications occurred in these patients during the TAVR that were unrelated to the blood sampling; ^2^ an arterial blood sample was taken instead of a venous blood sample.

This study included 50 patients who underwent TAVR due to aortic valve stenosis. The indication was independently approved by the interdisciplinary “Heart Team” of the University Hospital RWTH Aachen. Patients were monitored during the TAVR according to the institutional protocol, with an arterial catheter and a central venous catheter in the superior vena cava. Patients who did not meet these criteria were excluded (Figure 1). Due to missing or incorrectly labeled blood samples four patients had to be excluded. Finally, data of 50 patients have been analyzed. Based on the pre-procedural left ventricular ejection fraction (LV-EF) the cohort was stratified into two groups, as proposed by Muller et al. [18]. The “cardiac group” comprised 16 patients with an LV-EF ≤ 50%, while the “non-cardiac group” included 34 patients with an LV-EF > 50%. All TAVR, anesthesia protocols, and ICU treatments were conducted in accordance with established local standards.

### 2.2. Measurement and Data Collection

**Figure 2:**
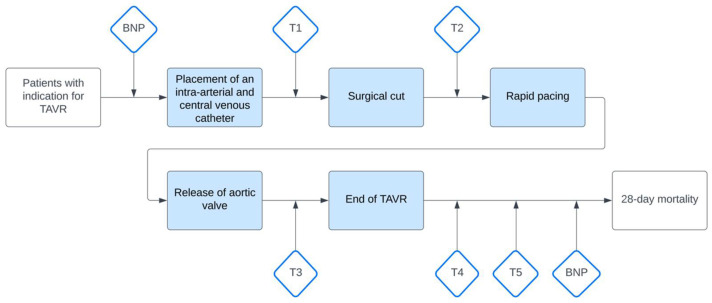
Study protocol. TAVR = transcatheter aortic valve replacement; NT-proBNP = N-terminal pro B-type natriuretic peptide; T1 = after central venous catheter placement, T2 = before rapid pacing, T3 = after valve release, T4 = end of TAVR, T5 = up to 2 h after TAVR.

Central venous (cv) and arterial (art) blood gases (cv pCO_2_, art pCO_2_, art pO_2_, cv pO_2_) were collected at specific time points: (T1) after the placement of the intra-arterial and central venous catheter, (T2) prior to rapid pacing, (T3) after valve deployment, (T4) at the end of the procedure, and (T5) within 2 h after the TAVR procedure (Figure 2). Blood samples were obtained simultaneously via the superior vena cava catheter and the intra-arterial catheter, both inserted prior to the procedure as stated above. The samples were analyzed immediately after collection using a blood gas analyzer (ABL90 FLEX, Radiometer, Copenhagen, Denmark).

Key parameters and patient characteristics were collected, including demographics, medical history, European System for Cardiac Operative Risk Evaluation II (EuroSCORE II) [22], Sequential Organ Failure Assessment (SOFA) score [23], NT-proBNP levels, hemodynamic data, blood gas values, and peri- and postoperative complications (detailed in Table 1 and Table 2, and the Appendix A and Appendix B Table A1 and Table A2). Major adverse cardiac events (MACE) were defined as the occurrence of acute myocardial infarction, cardiovascular death, unstable angina, or heart failure and were recorded as part of the complications.

Central-venous and arterial pCO_2_ was used to calculate the CO_2_ gap (mmHg). The difference between the central venous and arterial CO_2_ content was calculated using the Douglas equation as described by Mallat et al. [24]:

(1) $${∆CCO}_{2D}={CvCO}_{2D}-{CaCO}_{2D}$$

(2) $${CCO}_{2D}=Plasma\;{CCO}_{2}\times \left[1-0.0289\times \left(Hb\right)/\left(3.352-0.456\times {SO}_{2}\right)\times \left(8.142-pH\right)\right]$$

Δ*CCO*_2*D*_ = difference between venous-to-arterial CO_2_ content (mL/dL)

*CvCO*_2*D*_ = venous CO_2_ content (mL/dL)

*CaCO*_2*D*_ = arterial CO_2_ content (mL/dL)

*CCO*_2*D*_ = the formula was used to calculate venous CO_2_ content (*CvCO*_2*D*_) using values obtained from venous blood samples and arterial CO_2_ content (*CaCO*_2*D*_) using values obtained from arterial blood samples.

0.289 dL/g, 3.352, 0.456, 8.142 = constants defined by Douglas (0.289 dL/g—correction factor reflecting the influence of Hb on CO_2_ buffering capacity; 3.352—empirical constant accounting for the interaction between dissolved CO_2_ and its bound form; 0.456—factor representing the impact of arterial oxygen saturation (SO_2_) on CO_2_ carriage in the blood; 8.142—constant reflecting the influence of blood pH on CO_2_ equilibrium).

*Hb* = hemoglobin (g/dL), SO_2_ = arterial oxygen saturation (%)

*Plasma CCO_2_* = CO_2_ content (ml/dL); calculated with:

(3) $$Plasma\;{CCO}_{2}=2.226\times S\times Plasma\;{PCO}_{2}\times \left(1+10^{pH-pK^{\prime }}\right)$$

*S* = plasma CO_2_ solubility coefficient; calculated with:

(4) $$S=0.0307+\left[0.00057\times \left(37-T\right)\right]+\left[0.00002\times {\left(37-T\right)}^{2}\right],\;where\;T\;is\;37\;{}^{\circ}C$$

*pK′* = calculated with:

(5) $$pK^{\prime }=6.086+\left[0.042\times \left(7.4-pH\right)\right]+\left(\left(38-T\right)\times \left\{0.00472+\left[0.00139\times \left(7.4-pH\right)\right]\right\}\right)\;where\;T\;is\;37\;{}^{\circ}C$$

The O_2_ content and the ratio of CO_2_ gap to arterial-to-venous O_2_ content were calculated as described by Mallat et al. [24]:

(6) $$∆O_{2}=C_{a}O_{2}-C_{v}O_{2}$$

(7) $$C_{a}O_{2}=1.34\times Hb\times S_{a}O_{2}+0.0031\times P_{a}O_{2}\;$$

(8) $$C_{v}O_{2\;}=1.34\times Hb\times S_{v}O_{2}+0.0031\times P_{v}O_{2}$$

*∆O*_2_ = arterial-to-venous oxygen content

*C_a_O*_2_ = arterial oxygen content (mL/dL)

*C_v_O*_2_ = venous oxygen content (mL/dL)

*Hb* = hemoglobin (g/dL)

*S_a_O*_2_ = arterial oxygen saturation (%)

*S_v_O*_2_ = venous oxygen saturation (%)

### 2.3. Primary and Secondary Outcome

The primary outcome was defined as the 28-day survival in patients undergoing TAVR as predicted by the CO_2_ gap. Secondary outcomes included assessment of CO_2_ gap differences between the cardiac and non-cardiac groups, as well as between survivors and non-survivors. Furthermore, the correlation between the CO_2_ gap and lactate levels within each group was analyzed, along with the differences in CO_2_ content between the cardiac and non-cardiac groups, and between survivors and non-survivors.

### 2.4. Statistical Analysis

SPSS software Version 22 (SPSS Inc., Chicago, IL, USA) and GraphPad Prism software (Version 10.2.3. for macOS, GraphPad Software, Boston, MA, USA.) were used for statistical analysis and graph design. The graphical abstract was generated using BioRender software (Science Suite Inc., Toronto, Canada) [25]. A two-tailed *p*-value < 0.05 was considered statistically significant. All data are reported as mean (SD), median (25th Quartile; 75th Quartile) or number (%). Continuous variables were assessed for normality. Normally distributed data are presented as mean ± standard deviation (SD), whereas non-normally distributed data are reported as median with interquartile range (IQR: 25th–75th quartile). If inclusion of patients in an analysis was not possible it is indicated in the description below the table or figure.

The Shapiro–Wilk test was used to assess the normal distribution of metric variables. For non-normally distributed data, a Mann–Whitney-U test was used to assess significant differences between the two groups. Student’s *t*-test was used for normal distributed data. For dichotomous variables, Fisher’s test was used when one of the cells had a count below 10. For the patient’s outcome variables, baseline characteristics that exhibited a difference between the two groups of *p* < 0.1 were considered as potential covariates and were adjusted for using the Quade test for non-parametric data.

Parameters measured at different time points were analyzed using two-way ANOVA or the Friedman test, as appropriate. Two-way ANOVA was employed for parametric data, while the Friedman test was applied for non-parametric data. Post hoc testing was conducted using Tukey’s correction. The correlation between lactate levels and the CO_2_ gap was assessed separately for the cardiac group, the non-cardiac group, and survivors using the Spearman correlation.

The Receiver Operating Characteristic (ROC) curve was employed to evaluate the statistical power of the CO_2_ gap as a prognostic marker. The optimal threshold value was determined using the Youden Index, while the Area Under the Curve (AUC) provided information about the test’s performance, with a value of 1 representing perfect discrimination between positive and negative cases, and a value of 0 indicating no better performance than random chance.

## 3. Results

### 3.1. Baseline, Perioperative and Postoperative Clinical Characteristics

Baseline clinical characteristics, comorbidities, scores, and perioperative parameters were similar between the cardiac and non-cardiac groups (Table 1). However, there were significant differences in cardiac status. Patients of the cardiac group had significantly more often atrial fibrillation (12 (75%) vs. 14 (41.2%); *p* = 0.035), a lower left ventricular ejection fraction (LV-EF) (37.5% (IQR: 29.3–48.8%) vs. 60% (IQR: 57–60%); *p* < 0.001), and coronary artery disease (15 (93.8%) vs. 18 (52.9%); *p* = 0.004). Pre- and postoperative NT-proBNP levels were more than twice as high in the cardiac group compared to the non-cardiac group (2599 pg/mL (IQR: 1915–6154 pg/mL) vs. 1089 pg/mL (IQR: 479–1914 pg/mL); *p* = 0.001 and 2547 pg/mL (IQR: 957–7202 pg/mL) vs. 1180 pg/mL (IQR: 529–1944 pg/mL); *p* = 0.007, respectively). There was no difference in postoperative complications, except for the occurrence of postoperative infection, which occurred significantly more often in patients in the cardiac group compared to the non-cardiac group (7 (43.8%) vs. 5 (14.7%); *p* = 0.036). There was no significant difference in 28-day mortality or major adverse cardiac events (MACE) between the cardiac and non-cardiac group (Table 1). In addition, there was no significant difference in terms of Intensive Care Unit (ICU) mortality, ICU readmission and length of ICU and hospital stay (Table 2).

Baseline, perioperative, and postoperative characteristics of survivors and non-survivors are presented in Appendix A Table A1. The TAVR time was significantly shorter in the survivor group compared to the non-survivor group (56.1 min (±18.7 min) vs. 101.5 min (±36.1 min), *p* = 0.026). Major adverse cardiac events (MACE) occurred significantly more frequently among non-survivors (0 (0%) vs. 2 (100%), *p* < 0.001). Additionally, significant differences were observed in the incidence of non-MACE perioperative complications (2 (4.2%) vs. 2 (100%), *p* = 0.005), as well as in the proportion of patients requiring red blood cell transfusions during TAVR or the postoperative care period (8 (16.7%) vs. 2 (100%), *p* = 0.037). Blood gas measurements, clinical parameters, and use of vasopressors are presented in Appendix B Table A2 for all patients from T1 to T5.

### 3.2. Primary and Secondary Outcomes

#### 3.2.1. No Significant CO_2_ Gap Difference Between Cardiac and Non-Cardiac or Between Survivors and Non-Survivors

**Figure 3:**
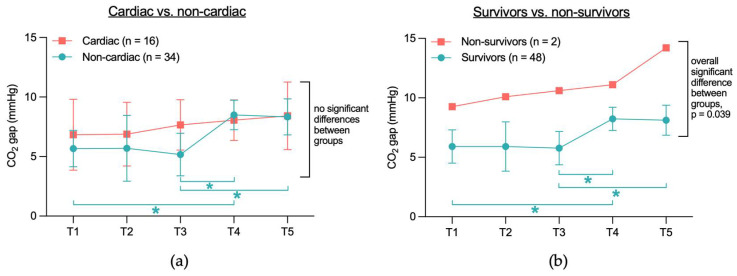
Temporal course of CO_2_ gap in (**a**) cardiac and non-cardiac patients and (**b**) survivors and non-survivors. Due to group size (*n* = 2), only mean is shown for non-survivors. CO_2_ gap = difference between central venous and arterial CO_2_ partial pressure; T1 = after placement of the central venous catheter, but before the start of the TAVR, T2 = immediately before rapid pacing, T3 = after aortic valve release, T4 = end of TAVR, T5 = up to 2 h after TAVR; * indicates that values differ significantly (*p* < 0.05) between two time points within survivors (blue).

When comparing the CO_2_ gap between the cardiac and non-cardiac groups between T1 to T5, no significant differences were found between the two groups (Figure 3a). However, analyzing CO_2_ gap values within each group across different time points, significant differences were observed in the non-cardiac group: T1 vs. T4 (5.7 mmHg vs. 8.5 mmHg, *p* = 0.008), T3 vs. T4 (5.2 mmHg vs. 8.5 mmHg, *p* = 0.031), and T3 vs. T5 (5.2 mmHg vs. 8.3 mmHg, *p* = 0.024). In contrast, the CO_2_ gap values in the cardiac group did not differ significantly across the time points T1 to T5.

When comparing the CO_2_ gap between survivors and non-survivors (Figure 3b), a significant overall difference was observed between the two groups (6.8 mmHg vs. 11.1 mmHg, *p* = 0.039). However, no significant differences were found when comparing the individual time points (*p* = 0.532). Within-group analysis revealed that in the survivor group, the CO_2_ gap values differed significantly at T1 vs. T4 (5.9 mmHg vs. 8.2 mmHg, *p* = 0.015), T3 vs. T4 (5.8 mmHg vs. 8.2 mmHg, *p* = 0.038), and T3 vs. T5 (5.8 mmHg vs. 8.1 mmHg, *p* = 0.047). In contrast, no significant differences were observed in the non-survivor group across time points T1 to T5.

#### 3.2.2. Limited Predictive Performance of CO_2_ Gap as a Prognostic Test for 28-Day Survival in Patients Undergoing a TAVR

To evaluate whether the CO_2_ gap can be used as a parameter to predict 28-day survival, a ROC analysis was performed. The overall model quality was determined as 0.53 at T1 and 0.52 at T4 (Table 3). ROC curves were not calculated for T2, T3, and T5 because the overall model quality at these time points was below 0.5, indicating no predictive ability beyond random chance.

**Figure 4:**
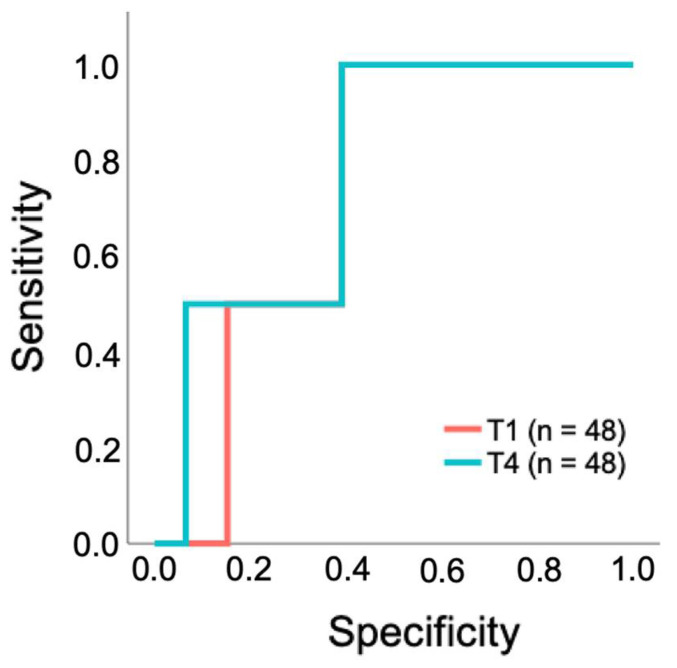
ROC curve for predicting 28-day survival based on CO_2_ gap at time points T1 and T4. ^1^ Due to missing 28-day follow-up data for 2 out of 50 patients, the analysis was conducted on the remaining 48 patients (*n* = 48 out of 50 patients). CO_2_ gap = difference between central venous and arterial CO_2_ partial pressure (mmHg); T1 = after placement of the central venous catheter, but before the start of the TAVR, T4 = end of TAVR.

At T1 and T4, the area under the curve (AUC) demonstrated moderate predictive performance (0.728 and 0.772, respectively). Threshold values were determined using the Youden index, with maximum CO_2_ gap of 7.3 mmHg at T1 and 8.7 mmHg at T4 (Table 3). The ROC curves in Figure 4 illustrate the predictive performance of the CO_2_ gap at these time points. The AUC values indicate moderate predictive performance; however, the ROC curve shows only limited discrimination between true-positive and false-positive rates (Figure 4).

According to the ROC analysis, cumulative mortality was compared at T1 with a cut-off of 7.3 mmHg and at T4 with a cut-off of 8.7 mmHg. The 28-day mortality rate in patients grouped by the CO_2_ gap threshold 7.3 mmHg at T1 was 10% (*n* = 2) for those with values >7.3mmHg (*n* = 20), while no death occurred in patients with values ≤7.3 mmHg (*n* = 28). However, this difference was not statistically significant (*p* = 0.168). At T4, patients grouped by the threshold of 8.7 mmHg showed a 28-day mortality rate of 5.3% (*n* = 1) for CO_2_ values > 8.7 mmHg (*n* = 19), compared to 3.5% (*n* = 1) in those with values ≤ 8.7 mmHg at T4 (*n* = 29). This difference was not statistically significant (*p* > 0.999).

#### 3.2.3. No Significant Correlation Between CO_2_ Gap and Lactate Levels in 28-Day Survivors and Non-Survivors, Cardiac or Non-Cardiac Group

To determine whether an association exists between CO_2_ gap values and lactate levels in survivors vs. non-survivors and cardiac vs. non-cardiac patients, correlation analyses were performed. No significant correlation was found between CO_2_ gap and lactate levels in the survivor group (Table 4). In the non-survivor group, Spearman correlation analysis could not be conducted due to limited sample size (*n* = 2 (4%)). Additionally, no significant correlation was observed between CO_2_ gap and lactate levels in the cardiac and non-cardiac groups (Table 5), except at T2, where a significant positive correlation was identified between lactate levels and CO_2_ gap in the cardiac group (Table 5a; *p* = 0.039, r = 0.525). The lactate measurements for all patients at each time point can be found in Appendix B Table A2.

**Figure 5:**
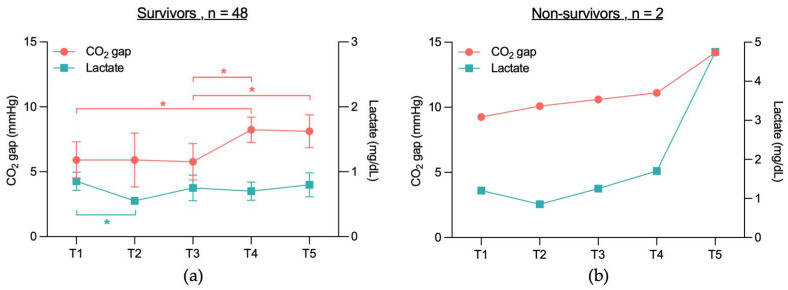
(**a**) CO_2_ gap vs. lactate levels in survivors at T1, T2, T3, T4 and T5. (**b**) CO_2_ gap vs. lactate in non-survivors at T1, T2, T3, T4 and T5. Due to the small group size of the non-survivor group (*n* = 2), only mean is shown. CO_2_ gap = difference between central venous and arterial CO_2_ partial pressure (mmHg); T1 = after placement of the central venous catheter, but before the start of the TAVR, T2 = immediately before rapid pacing, T3 = after aortic valve release, T4 = end of TAVR, T5 = up to 2 h after TAVR; * indicates that values differ significantly (*p* < 0.05) between two time points within lactate (blue) or CO_2_ gap (red).

When analyzing lactate levels in the survivor and non-survivor groups, a significant difference was observed in the survivor group between lactate levels at T1 and T2 (0.9 mg/dL vs. 0.6 mg/dL, *p* = 0.0001) (Figure 5a). In the non-survivor group, however, no significant differences in lactate levels were found across time points T1 to T5 (Figure 5b).

**Figure 6:**
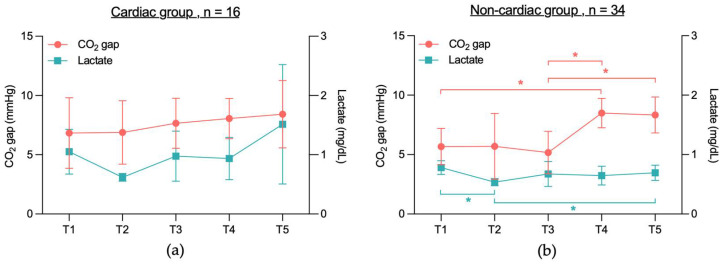
(**a**) CO_2_ gap vs. lactate levels in the cardiac group at T1, T2, T3, T4 and T5. (**b**) CO_2_ gap vs. lactate levels in the non-cardiac group at T1, T2, T3, T4 and T5. CO_2_ gap = difference between central venous and arterial CO_2_ partial pressure (mmHg); T1 = after placement of the central venous catheter, but before the start of the TAVR, T2 = immediately before rapid pacing, T3 = after aortic valve release, T4 = end of TAVR, T5 = up to 2 h after TAVR; * indicates that values differ significantly (*p* < 0.05) between two time points within lactate (blue) or CO_2_ gap (red).

Analyzing lactate levels in the cardiac and non-cardiac groups revealed no significant differences in lactate levels between time points T1 and T5 in the cardiac group (Figure 6a). In the non-cardiac group however, significant differences were observed between T1 and T2 (0.8 mg/dL vs. 0.5 mg/dL, *p* = 0.0004) and between T2 and T5 (0.5 mg/dL vs. 0.7 mg/dL, *p* = 0.026) (Figure 6b).

#### 3.2.4. Difference in CO_2_ Content Calculated with the Douglas Equation Between Cardiac and Non-Cardiac Group and Between Survivors and Non-Survivors

**Figure 7:**
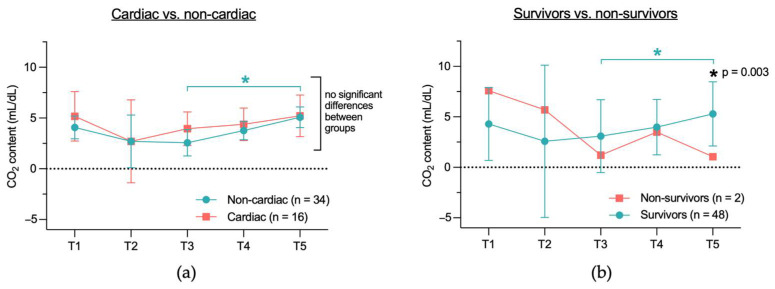
Temporal course of cv-art CO_2_ content in the (**a**) cardiac and non-cardiac group and (**b**) the survivor and non-survivor group. Cv-art CO_2_ content is measured with the Douglas equation. Due to the small group size of the non-survivor group (*n* = 2), only mean is shown. Cv-art CO_2_ content = difference between central venous and arterial CO_2_ content; T1 = after placement of the central venous catheter, but before the start of the TAVR, T2 = immediately before rapid pacing, T3 = after aortic valve release, T4 = end of TAVR, T5 = up to 2 h after TAVR; * indicates a significant difference (*p* < 0.05) between two time points (blue) or between two groups at a specific time point (black).

The central venous-to-arterial CO_2_ content (cv-art CO_2_) was calculated using the Douglas equation [24]. There was no significant difference in the cv-art CO_2_ content between the cardiac and non-cardiac groups (Figure 7a). When comparing the cv-art CO_2_ content between survivors and non-survivors, no significant differences were observed between the groups, except at T5 (5.3 mL/dL vs. 1.1 mL/dL, *p* = 0.003) (Figure 7b). Comparing the cv-art CO_2_ content across individual time points (T1–T5) within each group revealed a significant difference in the non-cardiac group between T3 and T5 (2.6 mL/dL vs. 5.1 mL/dL, *p* = 0.021) (Figure 7a) and in the survivor group between T3 and T5 (3.1 mL/dL vs. 5.3 mL/dL, *p* = 0.007) (Figure 7b).

#### 3.2.5. Ratio of CO_2_ Gap to Arterial-to-Venous O_2_ Content Difference in Survivors and Non-Survivors

**Figure 8:**
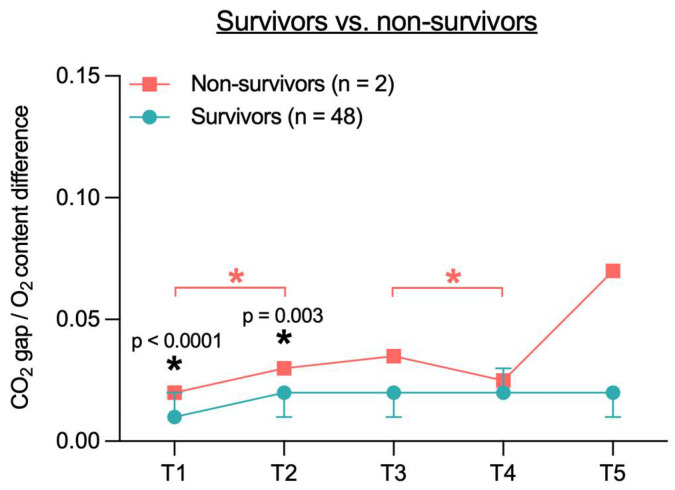
Ratio of CO_2_ gap to the arterial-to-venous O_2_ content difference in survivors and non-survivors at different time points. Due to the small group size of the non-survivor group (*n* = 2), only mean is shown. CO_2_ gap = difference between central venous and arterial CO_2_ partial pressure; T1 = after placement of the central venous catheter, but before the start of the TAVR, T2 = immediately before rapid pacing, T3 = after aortic valve release, T4 = end of TAVR, T5 = up to 2 h after TAVR; * indicates a significant difference (*p* < 0.05) between two time points (red) or between two groups at a specific time point (black).

When calculating the ratio of the CO_2_ gap to the arterial-to-venous O_2_ content difference (CO_2_ gap/O_2_ content difference) in survivors and non-survivors, no overall significant difference was observed (Figure 8). However, there was a significant difference between survivors and non-survivors at time point T1 (0.01 vs. 0.02, *p* <0.0001) and at T2 (0.02 vs. 0.03, *p* = 0.003). In addition, significant differences were observed between T1 and T2 (0.02 vs. 0.03, *p* < 0.0001) and between T3 and T4 (0.04 vs. 0.03, *p* < 0.0001) within the non-survivor group.

## 4. Discussion

The aim of this study was to evaluate the prognostic value of the CO_2_ gap for 28-day survival in patients undergoing a transcatheter aortic valve replacement (TAVR), and to analyze its association with cardiac function, markers of tissue perfusion such as lactate levels, and parameters of O_2_ and CO_2_ exchange, including the CO_2_ content and the CO_2_ gap/O_2_ content difference.

Baseline characteristics were largely comparable between the cardiac and non-cardiac groups, with the exception of a higher prevalence of atrial fibrillation, coronary artery disease, and elevated NT-proBNP levels in the cardiac group. Postoperative complication rates were similar between groups, although infections occurred more often in the cardiac cohort. Outcomes such as 28-day mortality, ICU mortality, ICU readmission, ICU length of stay, and hospital length of stay did not differ significantly between groups. Analysis of the CO_2_ gap revealed no significant difference between the cardiac and non-cardiac group. Non-survivors showed a significantly higher CO_2_ gap overall compared to survivors, although no significant differences were found at individual time points. Despite this finding, ROC curve analysis indicated only limited prognostic value of the CO_2_ gap for 28-day survival, and there were no statistically significant differences in 28-day mortality between patients above or below the CO_2_ gap thresholds at T1 and T4. Furthermore, no significant correlation between the CO_2_ gap and lactate levels was found in any group, except for a positive correlation at T2 within the cardiac group. CO_2_ content, calculated with the Douglas equation, did not differ significantly between cardiac and non-cardiac patients, nor were there consistent differences between survivors and non-survivors. Finally, while the CO_2_ gap/O_2_ content difference was significantly elevated in non-survivors at T1 and T2, this difference did not remain significant across all time points.

The CO_2_ gap has been associated with reduced survival and higher complication rates, particularly in patients with sepsis [18,26]. Although the underlying mechanisms are complex, an elevated CO_2_ gap is generally considered a sign of inadequate tissue perfusion [14,15]. Furthermore, the CO_2_ gap has been shown to inversely correlate with cardiac output [27], supporting its role as an indicator of low-blood flow states. It suggests that the CO_2_ gap may be a valuable marker, especially in the setting of cardiac surgery. To date, however, the CO_2_ gap has not been evaluated in patients with aortic valve stenosis or those undergoing a TAVR. While established markers such as elevated preprocedural [28] and postprocedural [29] troponin levels, as well as increased BNP levels [30], have been associated with worse outcomes after TAVR, there is currently limited evidence regarding periprocedural markers that reflect rapid physiological changes and predict long-term survival.

The results of this study demonstrate no significant prognostic value of the CO_2_ gap. In contrast, other studies have identified the CO_2_ gap as a valid prognostic marker for 28-day mortality in patients with septic shock [18,26] and have also shown an association with an increased rate of complications in patients undergoing high-risk abdominal and visceral surgeries, such as hepatectomy or septic surgery [20]. The elevation of the CO_2_ gap is the result of a complex interplay of multiple factors, including CO_2_ production, the CO_2_ dissociation curve, and changes in both micro- and macrovascular circulation [14,26,27]. Among these, insufficient blood flow appears particularly important, as it leads to CO_2_ accumulation and thus to an increased CO_2_ gap. This mechanism was demonstrated in septic patients [31], where targeted fluid resuscitation led to a decrease in the CO_2_ gap and an increase in cardiac output. Similarly, a low cardiac index [19,27] in septic shock has been associated with a higher CO_2_ gap, highlighting the role of hypoperfusion.

To assess the relationship between cardiac function and the CO_2_ gap, patients in the present study were classified into cardiac and non-cardiac groups, following the approach described by Muller et al. [18]. While Muller [18] reported that an increased CO_2_ gap was associated with impaired cardiac function and reduced survival in patients with septic shock, this study did not find a significant difference in the CO_2_ gap between the cardiac and non-cardiac groups.

Significantly elevated lactate levels in patients undergoing open heart surgery have been associated with low cardiac output and increased postoperative mortality, suggesting a potential prognostic value of lactate [32]. Furthermore, previous research demonstrated a correlation between an increased CO_2_ gap and elevated lactate levels [27]. In the present study, these findings could not be confirmed, which may be due to the small sample size. However, lactate levels can be influenced by many factors, which may limit their reliability as a marker of tissue perfusion. Although lactate is a well-established diagnostic and prognostic marker [33,34,35], its elevation is not specific to conditions such as shock, sepsis, or impaired cardiac function, but can also result from a variety of other factors. These include long-term beta-blocker therapy [36] as well as clinical conditions such as seizures, infections, acute abdominal pathology, or metabolic disorders [37]. Therefore, elevated lactate levels require further diagnostic evaluation and do not necessarily reflect impaired perfusion or reduced blood flow. In addition, lactate levels tend to respond more slowly to hemodynamic changes, and dynamic trends are oftentimes more useful than a single measurement [38]. An increased CO_2_ gap may better reflect perfusion abnormalities.

Furthermore, previous studies have also shown that CO_2_ levels correlate with CO_2_ content, although being influenced by several factors [24,39], including Hb concentration, the Haldane effect [40], haematocrit, and blood pH. These variables are incorporated into the calculation of CO_2_ content within the Douglas equation but are not considered in the calculation of the CO_2_ gap, suggesting that the calculation of the CO_2_ content may provide a more accurate reflection of the metabolic state than the calculation of the CO_2_ gap alone.

In the present study, however, no significant differences in CO_2_ content were observed between the cardiac and non-cardiac groups. Similarly, the CO_2_ content did not differ significantly between survivors and non-survivors, with the exception at time point T5, where survivors showed a higher CO_2_ content compared to non-survivors. This finding at T5 appears counterintuitive, as impaired perfusion in non-survivors would be expected to result in a higher CO_2_ content. The small sample size and potential inaccuracies in the CO_2_ content calculation may explain the lack of significant differences between groups, as well as the unexpected finding at T5. Although the Douglas equation incorporates multiple physiological variables to enhance precision, previous studies have described this calculation method as being prone to error [24,41], as these variables themselves are subject to dynamic fluctuations, causing discrepancies between the calculated and actual physiological values.

Several studies have also suggested the ratio of the CO_2_ gap to the arterial-to-venous O_2_ content difference may provide more reliable prognostic information than the CO_2_ gap alone [16]. Persistently elevated lactate levels in combination with an increased CO_2_ gap/O_2_ content difference have been associated with severe organ dysfunction and worse survival in septic patients [42]. One study also reported no significant association between the CO_2_ gap alone and survival, whereas the CO_2_ gap/O_2_ content difference more accurately predicted lactate trends and correlated with increased mortality [43]. This approach is based on the assumption that the ratio better reflects anaerobic metabolism [43] and thereby serves as an indirect marker of tissue oxygenation [14].

While an elevated CO_2_ gap primarily indicates hypoperfusion [40], the CO_2_ gap/O_2_ content difference is more sensitive to anaerobic conditions, regardless of perfusion status [16]. In hypoxic state, CO_2_ production (VCO_2_) increases relative to O_2_ consumption (VO_2_), resulting in a higher VCO_2_/VO_2_ ratio. According to the Fick principle, this leads to an increased CO_2_ gap/O_2_ content difference [14,16]. In the present study, no overall significant differences in this ratio were observed between the cardiac and non-cardiac groups. However, significant differences between survivors and non-survivors were found at T1 and T2. These findings may indicate a potential prognostic value of the CO_2_ gap/O_2_ content difference, which could become significant in a larger cohort.

In this study, the cardiac group had a significant higher rate of postoperative infections compared to the non-cardiac group. However, as most infections were diagnosed after the procedure, it is unlikely that they had an impact on the samples at T5, which were obtained two hours post-intervention. In the non-cardiac group, one patient developed aspiration-related pneumonia during TAVR, which could have affected gas exchange [44] and thereby potentially could have influenced the results.

This study has several limitations. As this was a pilot study, the sample size was limited, particularly in the non-survivor group. A larger cohort and a multicenter study design may help to detect significant differences. Due to the small sample size, even a limited number of events may have resulted in a comparatively high observed mortality rate. Therefore, these findings should be interpreted with caution, and larger studies are required to validate the results. Furthermore, alternative sampling times or an extended sampling period might have provided additional insights.

## 5. Conclusions

In this study, no clear evidence was found to support the CO_2_ gap as a reliable prognostic marker for 28-day survival in patients undergoing TAVR. Larger, multicenter studies involving broader patient populations are needed to further clarify the clinical relevance of the CO_2_ gap in patients undergoing a TAVR.

## Figures and Tables

**Table 1:** Baseline, perioperative and postoperative clinical characteristics of all patients, non-cardiac and cardiac groups.

Characteristic	All Patients *n* = 50	Non-Cardiac *n* = 34	Cardiac *n* = 16	*p*-Value
Baseline clinical characteristics				
Age (years)	82 (76–86)	82 (75–86)	83 (79–85)	0.851
Male/Female	30/20 (60%/40%)	17/17 (50%/50%)	13/3 (81.3%/18.8%)	0.062
BMI (kg/m^2^)	28.6 (25.7–31)	28.7 (24.8–31.7)	27.6 (25.8–30)	0.835
Comorbidities				
COPD	7 (14%)	5 (14.7%)	2 (12.5%)	>0.999
Pathological pulmonary function test ^1^	5 (10.4%)	3 (8.8%)	2 (14.3%)	0.621
Diabetes mellitus	17 (34%)	12 (35.3%)	5 (31.3%)	>0.999
Arterial hypertension	40 (80%)	27 (79.4%)	13 (81.3%)	>0.999
Liver cirrhosis	0 (0%)	0 (0%)	0 (0%)	>0.999
Chronic kidney disease	17 (34%)	10 (29.4%)	7 (43.8%)	0.353
Cardiac status at study entry				
Chronic heart failure	34 (68%)	21 (61.8%)	13 (81.3%)	0.208
Atrial fibrillation	26 (52%)	14 (41.2%)	12 (75%)	0.035 *
History of myocardial infarction	12 (24%)	6 (17.6%)	6 (37.5%)	0.163
LV-EF (%)	57.5 (48.8–60)	60 (57–60)	37.5 (29.3–48.8)	< 0.001 *
History of CABG surgery	6 (12%)	5 (14.7%)	1 (6.3%)	0.650
Coronary artery disease (CAD)	33 (66%)	18 (52.9%)	15 (93.8%)	0.004 *
Preoperative NT-proBNP level ^2^ (pg/mL)	1434 (678.5–2659)	1089 (479–1914)	2599 (1915–6154)	0.001 *
Scores				
SOFA	2 (0.8–2)	2 (0–2)	2 (1–2)	0.539
EuroSCORE II	3.1 (1.8–4.3)	2.8 (1.8–3.7)	3.4 (2.2–5.7)	0.114
Perioperative clinical characteristics				
Length of TAVR (min)	53.5 (42.3–67)	53 (42–63)	58 (45–72)	0.355
Patients with invasive ventilation during TAVR	3 (6%)	2 (5.9%)	1 (6.3%)	>0.999
All complications	14 (28%)	9 (26.5%)	5 (31.3%)	0.746
CPR	3 (6%)	1 (2.9%)	2 (12.5%)	0.237
Bleeding	5 (10%)	3 (8.8%)	2 (12.5%)	0.650
AV block III°	5 (10%)	5 (14.7%)	0 (0%)	0.163
Others ^3^	4 (8%)	2 (5.9%)	2 (12.5%)	0.584
Postoperative clinical characteristics				
Postoperative NT-proBNP level (pg/mL)	1493 (715–2498)	1180 (529–1944)	2547 (957–7202)	0.007 *
Patients with invasive ventilation during ICU stay	5 (10%)	3 (8.8%)	2 (12.5%)	0.650
Number of patients requiring red blood cell transfusion ^4^	10 (20%)	5 (14.7%)	5 (31.3%)	0.256
All complications	42 (84%)	27 (79.4%)	15 (93.8%)	0.409
Bleeding at the vascular access site or pseudoaneurysm	16 (32%)	10 (29.4%)	6 (37.5%)	0.746
Mild paravalvular leakage	16 (32%)	12 (35.3%)	4 (25%)	0.533
AV block III°	18 (36%)	13 (38.2%)	5 (31.3%)	0.757
Atrial fibrillation requiring therapy ^5^	4 (8%)	2 (5.9%)	2 (12.5%)	0.584
New permanent pacemaker implantation	18 (36%)	13 (38.2%)	5 (31.3%)	0.757
Other cardiac arrhythmias ^6^	19 (38%)	12 (35.3%)	7 (43.8%)	0.756
Acute kidney injury	6 (12%)	4 (11.8%)	2 (12.5%)	>0.999
Infection ^7^	12 (24%)	5 (14.7%)	7 (43.8%)	0.036 *
Stroke	1 (2%)	1 (2.9%)	0 (0%)	>0.999
Delirium	1 (2%)	1 (2.9%)	0 (0%)	>0.999
Others ^8^	18 (36%)	12 (35.3%)	6 (37.5%)	>0.999
Major adverse cardiac events (MACE) ^9^	2 (4%)	1 (2.9%)	1 (6.3%)	0.542

Results are expressed as median (first quartile–third quartile) or number (%); *p* < 0.05 was considered statistically significant (*). BMI = Body-Mass-Index; COPD = Chronic obstructive pulmonary disease; LV-EF = Left ventricular ejection fraction; CABG = Coronary artery bypass graft; CAD = Coronary artery disease; BNP = N-terminal pro B-type natriuretic peptide; SOFA = Sequential organ failure assessment score; EuroSCORE II = European system for cardiac operative risk Evaluation II; TAVR = transcatheter aortic valve replacement; CPR = cardiopulmonary resuscitation; AV block = atrioventricular block; ICU = intensive care unit; MACE = major adverse cardiac event. ^1^ 2 of 48 patients did not undergo a preoperative pulmonary function test; ^2^ preoperative NT-proBNP levels could not be obtained for 1 out of 50 patients in the cardiac group due to missing blood sample; ^3^ other complications: one patient in the cardiac and one in the non-cardiac group required red blood cell transfusion, one patient in the non-cardiac group required intubation due to aspiration and in one patient from the cardiac group, a second valve had to be inserted due to the first valve being dislocated; ^4^ red blood cell transfusion both during TAVR and during postoperative care period are included; ^5^ therapies included electrocardioversion or administration of amiodarone; ^6^ including right bundle branch block (RBBB), left bundle branch block (LBBB), left anterior hemiblock (LAHB), first degree atrioventricular block (AVB); ^7^ a total of five infections were recorded in the non-cardiac group, including one case of urinary tract infection, two cases of pneumonia—one of which was aspiration-related and occurred during the TAVR procedure—and two infections with an unknown focus. Seven infections were recorded in the cardiac group, including one case of pneumonia, two cases of urinary tract infection, and four infections of unknown etiology. All infections were diagnosed after the TAVR procedure, with the exception of the aspiration pneumonia, which occurred during the intervention. ^8^ other complications: 10 patients required red blood cell transfusion, one patient had a pulmonary embolism, one patient had a Stanford A aortic dissection, one patient had a cardiac tamponade, one patient was readmitted to the hospital due to cardiac decompensation, one patient had a hemodynamic irrelevant pericardiac effusion, three patients developed hematological changes, two patients discharged themselves, one patient had a dislocation of the pacemaker probe, two patients had unclear drop in hemoglobin, one patient had a follow-up due to hematoma, one patient developed a contrast medium-induced hyperthyroidism; ^9^ MACE includes complications such as acute myocardial infection, cardiovascular death, unstable angina and heart failure. Two patients suffered cardiovascular deaths.

**Table 2:** Patients’ outcomes in cardiac and non-cardiac group.

Characteristic	All Patients *n* = 50	Non-Cardiac *n* = 34	Cardiac *n* = 16	*p*-Value	ANOVA Adjusted *p*-Value ^1^
28-day mortality ^2^	2 (4.2%)	1 (3.1%)	1 (6.3%)	0.560	0.796
ICU mortality	2 (4%)	1 (2.9%)	1 (6.3%)	0.542	0.876
ICU readmission	2 (4%)	2 (5.9%)	0 (0%)	0.458	0.297
ICU LOS (days)	1 (1–2)	1 (1–2)	1 (1–1.8)	0.928	0.687
Hospital LOS (days)	10.5 (7.5–2)	9 (6.8–21)	14 (9.3–21)	0.172	0.309

Results are expressed as median (first quartile–third quartile) or number (%). ICU = intensive care unit; LOS = length of stay. ^1^ Analysis was adjusted for baseline characteristics with a *p*-value < 0.1 (gender, atrial fibrillation, CAD (= coronary artery disease) and preoperative BNP (= brain natriuretic peptide)); ^2^ Due to missing 28-day follow-up data for 2 out of 50 patients, the values were only calculated for 48 patients.

**Table 3:** Diagnostic parameters for CO_2_ gap in predicting 28-day survival at time points T1 and T4 (*n* = 48 patients with survival status available at 28 days).

Clinical Variable	Overall Model Quality	Optimal Cutoff	Youden Index	Sensitivity	Specificity	AUC
T1 CO_2_ gap	0.53	7.3 mmHg	0.609	100	61	0.728
T4 CO_2_ gap	0.52	8.7 mmHg	0.609	100	61	0.772

Due to missing 28-day follow-up data for 2 out of 50 patients, the analysis was conducted on the remaining 48 patients (*n* = 48 out of 50 patients). CO_2_ gap = difference between central venous and arterial CO_2_ partial pressure (mmHg); AUC = area under the curve; T1 = after placement of the central venous catheter, but before the start of the TAVR, T4 = end of TAVR.

**Table 4:** Spearman correlation analysis for CO_2_ gap vs. lactate levels in survivors.

Survivors Group	*p*-Value	Spearman r
CO_2_ gap T1 vs. lactate T1	0.103	0.238
CO_2_ gap T2 vs. lactate T2	0.778	0.042
CO_2_ gap T3 vs. lactate T3	0.329	−0.144
CO_2_ gap T4 vs. lactate T4	0.957	−0.008
CO_2_ gap T5 vs. lactate T5	0.746	−0.048

CO_2_ gap = difference between central venous and arterial CO_2_ partial pressure (mmHg); T1 = after placement of the central venous catheter, but before the start of the TAVR, T2 = immediately before rapid pacing, T3 = after aortic valve release, T4 = end of TAVR, T5 = up to 2 h after TAVR.

**Table 5:** Spearman correlation analysis for CO_2_ gap vs. lactate levels in (a) the cardiac group and in (b) the non-cardiac group.

**a: Cardiac group**	***p*** **-Value**	**Spearman r**
CO_2_ gap T1 vs. lactate T1	0.121	0.404
CO_2_ gap T2 vs. lactate T2	0.039 *	0.525
CO_2_ gap T3 vs. lactate T3	0.720	−0.096
CO_2_ gap T4 vs. lactate T4	0.705	0.102
CO_2_ gap T5 vs. lactate T5	0.422	0.214
**b: Non-cardiac group**	***p*** **-Value**	**Spearman r**
CO_2_ gap T1 vs. lactate T1	0.367	0.160
CO_2_ gap T2 vs. lactate T2	0.679	−0.074
CO_2_ gap T3 vs. lactate T3	0.205	−0.223
CO_2_ gap T4 vs. lactate T4	0.714	−0.065
CO_2_ gap T5 vs. lactate T5	0.859	−0.032

CO_2_ gap = difference between central venous and arterial CO_2_ partial pressure (mmHg); T1 = after placement of the central venous catheter, but before the start of the TAVR, T2 = immediately before rapid pacing, T3 = after aortic valve release, T4 = end of TAVR, T5 = up to 2 h after TAVR; * *p* < 0.05 was considered statistically significant (*).

## Data Availability

The datasets generated and analyzed during the current study are not publicly available due to privacy and ethical restrictions. Anonymized data may be made available from the corresponding author upon reasonable request.

## Abbreviations

The following abbreviations are used in this manuscript:

CO_2_ gap	venous-to-arterial difference in partial pressure of carbon dioxide
TAVR	transcatheter aortic valve replacement
LV-EF	left ventricular ejection fraction
ICU	intensive care unit
SOFA Score	Sequential organ failure assessment
EuroSCORE II	European system for cardiac operative risk evaluation II
MACE	major adverse cardiac events
NT-proBNP	N-terminal pro B-type natriuretic peptide
ROC	Receiver Operating Characteristics
AUC	Area Under the Curve

## Appendix A

**Table A1:** Baseline, perioperative and postoperative clinical characteristics of survivors and non-survivors.

Characteristic	Survivor (*n* = 48)	Non-Survivor (*n* = 2)	*p*-Value
Baseline clinical characteristics			
Age (years)	80 ± 6.4	87 ± 4.2	0.132
Male/Female	29/19 (60.4%/39.6%)	1/1 (50%/50%)	>0.999
BMI (kg/m^2^)	28.7 ± 4.4	30.2 ± 8.8	0.643
Comorbidities			
COPD	7 (14.6%)	0 (0%)	>0.999
Pathological pulmonary function test ^1^	5 (10.9%)	0 (0%)	>0.999
Diabetes mellitus	16 (33.3%)	1 (50%)	>0.999
Arterial hypertension	38 (79.2%)	2 (100%)	>0.999
Liver cirrhosis	0 (0%)	0 (0%)	
Chronic kidney disease	16 (33.3%)	1 (50%)	>0.999
Cardiac status at study entry			
Chronic heart failure	34 (70.8%)	0 (0%)	0.098
Atrial fibrillation	25 (52.1%)	1 (50%)	>0.999
History of myocardial infarction	12 (25%)	0 (0%)	>0.999
LV-EF (%)	52.5 ± 10.9	43 ± 24	0.589
History of CABG surgery	5 (10.4%)	1 (50%)	0.228
Coronary artery disease (CAD)	31 (64.6%)	2 (100%)	0.542
Preoperative NT-proBNP level ^2^ (pg/mL)	2272.3 ± 2893.6	3233 ± 1422.7	0.206
Scores			
SOFA	1.4 ± 1	2.5 ± 0.7	0.147
Euro II Score	4 ± 3.8	9.5 ± 7.1	0.080
Perioperative clinical characteristics			
Length of TAVR (minutes)	56.1 ± 18.7	101.5 ± 36.1	0.026 *
Patients with invasive ventilation during TAVR	3 (6.3%)	0 (0%)	>0.999
All complications	12 (25%)	2 (100%)	0.074
CPR	3 (6.3%)	0 (0%)	>0.999
Bleeding	4 (8.3%)	1 (50%)	0.192
AV block III°	5 (10.4%)	0 (0%)	>0.999
Others ^3^	2 (4.2%)	2 (100%)	0.005 *
Postoperative clinical characteristics			
Postoperative NT-proBNP level (pg/mL)	2547.1 ± 3448.3	1856.5 ± 381.1	0.558
Patients with invasive ventilation during ICU stay	4 (8.3%)	1 (50%)	0.192
Number of patients requiring red blood cell transfusion ^4^	8 (16.7%)	2 (100%)	0.037 *
All complications	40 (83%)	2 (100%)	>0.999
Bleeding at the vascular access site or pseudoaneurysm	15 (31.3%)	1 (50%)	0.542
Mild paravalvular leakage	16 (33.3%)	0 (0%)	>0.999
AV block III°	8 (16.7%)	0 (0%)	>0.999
Atrial fibrillation requiring therapy ^5^	4 (8.3%)	0 (0%)	>0.999
New permanent pacemaker implantation	18 (37.5%)	0 (0%)	0.530
Other cardiac arrhythmias ^6^	19 (39.6%)	0 (0%)	0.519
Acute kidney injury	6 (12.5%)	0 (0%)	>0.999
Infection	12 (25%)	0 (0%)	>0.999
Stroke	1 (2.1%)	0 (0%)	>0.999
Delirium	1 (2.1%)	0 (0%)	>0.999
Others ^7^	16 (33.3%)	2 (100%)	0.125
Major adverse cardiac events (MACE) ^8^	0 (0%)	2 (100%)	<0.001 *

Results are expressed as number (%); *p* < 0.05 was considered statistically significant (*). Due to the small group size of the non-survivor group (*n* = 2), metric variables are expressed as mean (standard deviation), despite not being normally distributed. BMI = Body Mass Index; COPD = Chronic obstructive pulmonary disease; LV-EF = Left ventricular ejection fraction; CABG = Coronary artery bypass graft; CAD = Coronary artery disease; NT-proBNP = N-terminal pro B-type natriuretic peptide; SOFA = Sequential organ failure assessment score; Euro II Score = European system for cardiac operative risk Evaluation; TAVR = transcatheter aortic valve replacement; CPR = cardiopulmonary resuscitation; AV block = atrioventricular block; ICU = intensive care unit; MACE = major adverse cardiac event. ^1^ 2 of 48 patients did not undergo a preoperative pulmonary function test.; ^2^ preoperative NT-proBNP levels could not be obtained for 1 out of 50 patients in the cardiac group due to missing blood sample; ^3^ other complications: two patients in the non-survivor group required red blood cell transfusion, one patient in the survivors group required intubation due to aspiration and in one patient from the survivors group, a second valve had to be inserted due to the first valve being dislocated; ^4^ red blood cell transfusion both during TAVR and during postoperative care period are included; ^5^ therapies included electrocardioversion or administration of amiodarone; ^6^ including right bundle branch block (RBBB), left bundle branch block (LBBB), left anterior hemiblock (LAHB), first degree atrioventricular block (AVB); ^7^ other complications: 10 patients required red blood cell transfusion, one patient had a pulmonary embolism, one patient had a Stanford A aortic dissection, one patient had a cardiac tamponade, one patient was readmitted to the hospital due to cardiac decompensation, one patient had a hemodynamic irrelevant pericardiac effusion, three patients developed hematological changes, two patients discharged themselves, one patient had a dislocation of the pacemaker probe, two patients had unclear drop in hemoglobin, one patient had a follow-up due to hematoma, one patient developed a contrast medium-induced hyperthyroidism; ^8^ MACE includes complications such as acute myocardial infection, cardiovascular death, unstable angina and heart failure. Two patients suffered cardiovascular deaths.

## Appendix B

**Table A2:** Blood gas measurements, clinical parameters, and vasopressors at each time point for all patients.

	T1	T2	T3	T4	T5	*p*-Value
Arterial CO_2_ pressure (mmHg)	42 (36.6–46.2)	48.5 (41.2–52.3)	48.2 (43.2–52.1)	44.7 (5.7)	41.9 (38–47.2)	<0.001 *
Central venous CO_2_ pressure (mmHg)	47.8 (43.9–51.5)	54.3 (49.4–57.8)	54.2 (49.6–57.1)	52.1 (49.6–55.4)	49.7 (46.1–53.2)	<0.001 *
SaO_2_ (%)	99.4 (98.1–100.1)	99.9 (99.6–100.2)	99.8 (99.1–100.1)	99.8 (98.9–100.2)	96.6 (94.3–98.3)	<0.001 *
SvO_2_ (%)	71.5 (8.7)	74 (7.5)	76.9 (9.3)	75.7 (69.1–80.4)	64.2 (8.7)	<0.001 *
aHb (g/dL)	12.3 (1.7)	11.3 (1.6)	10.7 (1.5)	10.6 (1.6)	11.5 (1.8)	<0.001 *
vHb (g/dL	12.3 (1.7)	11.2 (1.7)	10.6 (1.5)	10.6 (1.7)	11.7 (1.7)	<0.001 *
Lactate (mg/dL)	0.8 (0.6–1)	0.6 (0.5–0.6)	0.6 (0.5–0.7)	0.6 (0.5–0.7)	0.6 (0.5–0.8)	<0.001 *
SpO_2_ (%)	100 (98.8–100)	100 (99–100)	100 (99–100)	99.5 (98–100)	96 (93.8–98)	<0.001 *
HR (bpm)	64.5 (61–74.3)	73 (16)	74 (63.8–89.5)	69.5 (60–78)	60.5 (55–69.3)	<0.001 *
MAP (mmHg)	87 (76–95)	75 (13)	79 (14)	73 (11)	72 (11)	<0.001 *
CVP ^1^ (mmHg)	12 (6)	15 (3)	17 (4)	12 (8–18)	10 (4)	<0.001 *
O_2_ gas flow rate (L/min)	6 (5.4–6)	6 (6–6)	6 (6–6)	6 (6–6)	2 (0.8–2)	<0.001 *
Infusion rate of norepinephrine (μg/kg/min)	0.000 (0.0000–0.0000)	0.0000 (0.0000–0.0324)	0.0000 (0.0000–0.0000)	0.0000 (0.0000–0.0235)	0.0000 (0.0000–0.0000)	<0.001 *
Infusion rate of epinephrine (μg/kg/min)	0.0000 (0.0000–0.0000)	0.0000 (0.0000–0.0000)	0.0000 (0.0000–0.0000)	0.0000 (0.0000–0.0000)	0.0000 (0.0000–0.0000)	0.713

Results are expressed as median (first quartile–third quartile), mean (standard deviation) or number (%); *p* < 0.05 was considered statistically significant (*). T1 = after placement of the central venous catheter, but before start of TAVR, T2 = immediately before rapid pacing, T3 = after aortic valve release, T4 = end of TAVR, T5 = up to 2 h after TAVR; SaO_2_ = arterial oxygen saturation; SvO_2_ = central-venous oxygen saturation; aHb = arterial hemoglobin; vHb = venous hemoglobin; SpO_2_ = saturation of peripheral oxygen; HR = heart rate; MAP = mean arterial pressure; CVP = central vein pressure. ^1^ CVP could not be collected for 25 out of 50 patients for T5 due to missing data.
